# Draft Genome Sequence of Staphylococcus pseudintermedius Strain 13-13613, Isolated from a Case of Canine Pyoderma

**DOI:** 10.1128/MRA.00027-20

**Published:** 2020-02-13

**Authors:** Inga Eichhorn, Antina Lübke-Becker, Antje-Maria Lapschies, Megan Jacob, Wolfgang Bäumer, Marcus Fulde

**Affiliations:** a Institute of Microbiology and Epizootics, Centre for Infection Medicine, Freie Universität Berlin, Berlin, Germany; b Department of Population Health and Pathobiology, College of Veterinary Medicine, NC State University, Raleigh, North Carolina, USA; c Institute of Pharmacology and Toxicology, Faculty of Veterinary Medicine, Freie Universität Berlin, Berlin, Germany; University of Rochester School of Medicine and Dentistry

## Abstract

Here, we report the draft genome sequence of Staphylococcus pseudintermedius strain 13-13613, isolated from a case of canine pyoderma. The draft genome contains 2,533,486 bp in 570 contigs.

## ANNOUNCEMENT

Staphylococcus pseudintermedius is a pathobiont that frequently colonizes the skin and the mucosal surfaces of dogs (1–4). As an opportunistic pathogen, S. pseudintermedius is a leading cause of canine pyoderma (5–7).

In a recent study, the first standardized *in vivo* model of S. pseudintermedius-mediated pyoderma in dogs was established, reflecting almost entirely the clinical syndrome observed in naturally occurring infections. Here, we present the draft genome sequence of this particular isolate (13-13613), which was originally isolated from a dog in North Carolina suffering from superficial pyoderma (8). These data will now allow both in-depth investigation of mechanisms underlying the pathogenesis of S. pseudintermedius-mediated pyoderma and testing and evaluation of therapeutic strategies in the standardized canine *in vivo* model.

S. pseudintermedius 13-13613 was grown in brain heart infusion (BHI) broth (Difco) overnight at 37°C. Bacterial DNA was isolated using the MasterPure complete DNA and RNA purification kit (Epicentre, Madison, WI, USA). Sequencing libraries were constructed using the Nextera XT library preparation kit according to the manufacturer’s instructions (Illumina, Inc., San Diego, CA, USA). Afterward, 300-bp paired-end reads were generated using an Illumina MiSeq sequencer. A total of 744,914 raw reads were generated for this library, and the whole sequencing run yielded a quality score (Q_30_) of ≥75.17%. The reads were *de novo* assembled into contigs with a minimum size of 200 bp using MIRA 4.0 (9) at default settings before the raw reads were (i) trimmed and filtered, (ii) error corrected and normalized, (iii) set as paired reads by name, and (iv) merged into paired reads using Geneious 10.1.3 with default settings. A total of 570 contigs were generated, ranging from 210 bp to 57,454 bp, with an *N*_50_ length of 13,630 bp, resulting in a total genome size of 2,533,486 bp. The cumulative G+C content of the genome assembly was 38.4%. Gene annotation was performed using the NCBI Prokaryotic Genome Annotation Pipeline (PGAP) 4.9, which predicted 2,532 coding DNA sequences, 67 tRNAs, and 8, 9, and 13 (5S, 16S, and 23S, respectively) rRNAs in the draft genome (10). As expected, classical staphylococcal virulence factors, such as coagulase (Coa), staphylococcal protein A (SPA), fibronectin-binding protein (FnbB), and secreted von Willebrand factor-binding protein (VWbp), were detected, whereas the genes encoding exfoliate toxins A and B (*expA* and *expB*, respectively) were absent from the annotated genome. S. pseudintermedius 13-13613 was assigned to the new sequence type 1323 (ST1323), due to a new *purA* allele, using the pubMLST database for S. pseudintermedius multilocus sequence typing (MLST) (Spm) (https://pubmlst.org/spseudintermedius/). Using ResFinder (11) and PlasmidFinder (12) (accessed at http://www.genomicepidemiology.org/ in August 2019) with default settings, neither resistance genes nor plasmids were identified. However, antimicrobial susceptibility testing using broth microdilution according to the Clinical and Laboratory Standards Institute (CLSI) recommendations revealed resistance to penicillin and ampicillin with MIC values of 8 mg/liter and 1 mg/liter, respectively (13). Consistently, the *fmtA* gene, encoding a low-affinity penicillin-binding protein, was detected with *in silico* analysis (14).

### Data availability.

This whole-genome shotgun project has been deposited at DDBJ/ENA/GenBank under the accession number VSRX000000000. The version described in this paper is version VSRX01000000. The raw data are available from the Sequence Read Archive (SRA) under the accession number SRX6750221.
